# Towards robust *Pseudomonas* cell factories to harbour novel biosynthetic pathways

**DOI:** 10.1042/EBC20200173

**Published:** 2021-07-26

**Authors:** Nora Lisa Bitzenhofer, Luzie Kruse, Stephan Thies, Benedikt Wynands, Thorsten Lechtenberg, Jakob Rönitz, Ekaterina Kozaeva, Nicolas Thilo Wirth, Christian Eberlein, Karl-Erich Jaeger, Pablo Iván Nikel, Hermann J. Heipieper, Nick Wierckx, Anita Loeschcke

**Affiliations:** 1 Institute of Molecular Enzyme Technology, Heinrich-Heine-University, Düsseldorf, Germany; 2 Institute of Bio- and Geosciences IBG-1: Biotechnology, Forschungszentrum Jülich, Germany; 3 The Novo Nordisk Foundation Center for Biosustainability, Technical University of Denmark, Lyngby, Denmark; 4 Department of Environmental Biotechnology, Helmholtz Centre for Environmental Research (UFZ), Leipzig, Germany

**Keywords:** biotechnology, chemical stress tolerance, host engineering, pseudomonas putida

## Abstract

Biotechnological production in bacteria enables access to numerous valuable chemical compounds. Nowadays, advanced molecular genetic toolsets, enzyme engineering as well as the combinatorial use of biocatalysts, pathways, and circuits even bring new-to-nature compounds within reach. However, the associated substrates and biosynthetic products often cause severe chemical stress to the bacterial hosts. Species of the *Pseudomonas* clade thus represent especially valuable *chassis* as they are endowed with multiple stress response mechanisms, which allow them to cope with a variety of harmful chemicals. A built-in cell envelope stress response enables fast adaptations that sustain membrane integrity under adverse conditions. Further, effective export machineries can prevent intracellular accumulation of diverse harmful compounds. Finally, toxic chemicals such as reactive aldehydes can be eliminated by oxidation and stress-induced damage can be recovered. Exploiting and engineering these features will be essential to support an effective production of natural compounds and new chemicals. In this article, we therefore discuss major resistance strategies of Pseudomonads along with approaches pursued for their targeted exploitation and engineering in a biotechnological context. We further highlight strategies for the identification of yet unknown tolerance-associated genes and their utilisation for engineering next-generation *chassis* and finally discuss effective measures for pathway fine-tuning to establish stable cell factories for the effective production of natural compounds and novel biochemicals.

## Introduction

Microbial biotechnology can provide chemical compounds that are essential for modern societies in multiple sectors, e.g. as pharmaceuticals and chemical building blocks. However, efficient microbial production using whole (living) cells requires a host that can cope with the associated stress. Aside from temperature or osmotic stress, this includes severe chemical stress caused by high concentrations of substrates and products needed to establish economically viable processes.

Bacteria have evolved numerous strategies to alleviate chemical stress and members of the *Pseudomonas* clade are especially well-equipped with such traits [1]. This has likely contributed to the development of the soil bacterium *Pseudomonas putida* and its relatives into versatile microbial cell factories during the past few decades, enabling the biosynthesis of various compounds including secondary metabolites like rhamnolipids, terpenes, polyketides, and non-ribosomal peptides, organic acids, alcohols, and aromatics [2–4]. Most of these products are not natively synthesised by the host strain, thus confronting cells with novel and – in parts – harmful chemistry, as many hydrophobic and antibiotic products tend to corrupt enzyme or membrane integrity. The *Pseudomonas* clade thus appears to provide an intriguing starting point to shed light on xenobiotic tolerance in the context of biotechnological applications such as plastics upcycling [5], aromatics production [4], and *trans*-metabolism [6].

Nowadays, even the production of new-to-nature compounds with potentially advantageous properties is coming into reach via engineering and combinatorial use of enzymes, pathways, and hosts, or hybrid bio- and chemical synthesis [7,8]. For example, the strain *P. putida* KT2440 was artificially equipped with parts of the polyketide synthase-/non-ribosomal peptide synthetase-type pathways for coronatine and prodigiosin biosynthesis to provide platforms that could incorporate supplemented unnatural amino acids or pyrrole precursors into new small molecules [9–11]. Another recent example challenged *P. putida* with xenobiotic organofluorine metabolites by use of a *Streptomyces* fluorinase and purine nucleotide phosphorylase for the synthesis of fluoronucleotides and fluorosugars [12].

The host’s robustness is evidently becoming a central aspect in strain development in the face of increasingly demanding production processes and targeted chemistry. Pseudomonads may benefit from their built-in characteristics when utilised as hosts [13–16]. For example, the solvent-tolerant *P. putida* S12 outperformed *E. coli* in the production of *p*-hydroxystyrene in biphasic cultures [17]. However, targeted engineering will likewise be necessary, especially for processes involving new-to-*Pseudomonas* compounds. The known robustness-associated features of different *Pseudomonas* strains can be assigned to three major strategies: (i) keeping stressors out of the cell by reducing permeability, (ii) eliminating stressors in the cell by export or conversion, and (iii) recovery of damaged structures. The molecular bases of these strategies have been comprehensively reviewed before with emphasis on organic solvent tolerance [18,19]. In this article, we discuss these leading tolerance and resistance strategies against a broader range of chemical stressors and their targeted exploitation in *Pseudomonas* platform strains along with future application perspectives (see Box 1 for key terms and concepts).

Box 1 **Key terms and concepts**

***Chassis*, host:** A microorganism with specific metabolic and robustness abilities (native or engineered), which allow functioning and purposeful utilisation.
**Cell factory:** An engineered *chassis*, which is utilised for biosynthesis of a product.
**Engineering:** Targeted or untargeted genetic modification aiming to adapt *chassis* abilities or cell factory performance.
**Bioproduction:** Biosynthesis of a product in an organism.
***Pseudomonas* bioproduction strains:** We focus on *P. putida* KT2440, *P. putida* S12, *P. putida* DOT-T1E, and *P. taiwanensis* VLB120 (because these are the hosts most widely described in the literature centred on chemical stress tolerance as reviewed in this article). Notably, the taxonomic status of *P. putida* strains is debated – with implications on their safety status. While strain KT2440 is generally accepted to be non-pathogenic and has HV1 status certified by the U.S. Food and Drug Administration (FDA) [20], regulatory matters for all other strains of the species are inconsistent, hampering biotechnological exploitation. A taxonomic revision of the *P. putida* clade has been suggested that proposed a new species *P. alloputida* [21], which encompasses the above-mentioned strains and distinguishes them from other species within the *P. putida* group including clinical isolates. Here, we refer to this proposed species as *P. putida* for the sake of consistency with prior literature.
**HV1 status:** A strain (together with a plasmid), which is certified as host–vector (HV) system safety level 1 (i.e. HV1), can be handled in a P1 (or biosafety level 1) facility.
**Resistance, tolerance, robustness:** Ability of an organism to withstand adverse conditions (this article’s focus lies on chemical compounds). The mechanistic differences that can be assigned to the terms [22] are usually not resolved in biotechnological research; here, we use these terms interchangeably.
  **Stressor:** An environmental condition (pH, temperature, ionic strength etc.) or a chemical compound (organic solvent, toxic substance etc.), that causes cellular and/or metabolic stress resulting, e.g., in impaired growth or cell death.
**Toxicant:** A toxic chemical compound, which can act as a causative agent of chemical stress (i.e. as stressor) in an organism via diverse mechanisms.
**Chemical stress:** Summary of adverse effects exerted by chemicals including corruption of membrane integrity, damage of macromolecules (DNA and proteins), and interference with metabolism (e.g. inhibition of protein biosynthesis).
**Xenobiotic:** A chemical compound foreign to an organism or an ecological system.
**New-to-*Pseudomonas* compound:** A xenobiotic to a *Pseudomonas* host.
**New-to-nature compound:** A chemical compound, which does not occur naturally.

## How Pseudomonads deal with toxic chemicals

In this chapter, we highlight the prominent natural stress responses of Pseudomonads, which allow them to sustain cell envelope integrity in the presence of toxicants, and to effectively export or eliminate them. We discuss the usefulness of these features for biotechnological applications and point out approaches for their targeted exploitation and engineering.

### Built-in cell envelope stress response as support of bioproduction

**Figure 1:**
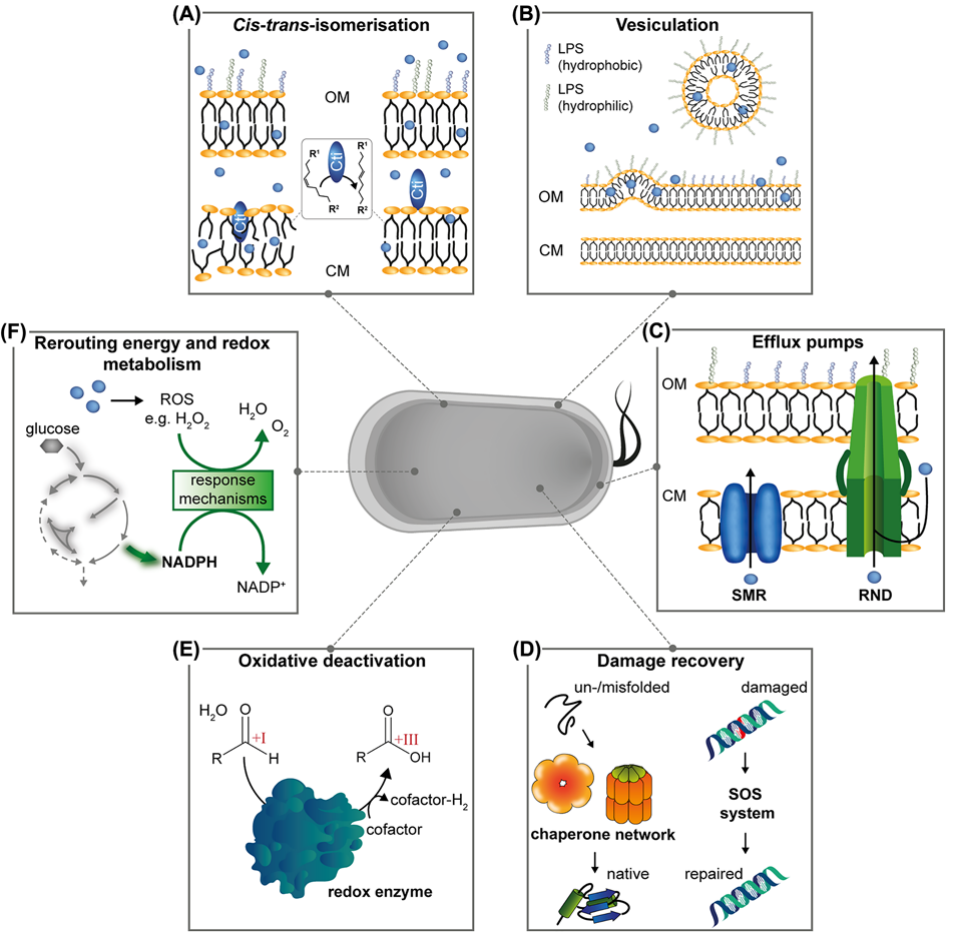
*Pseudomonas* natural stress response mechanisms against harmful chemicals Bacteria counter chemical stress (blue spheres) with different mechanisms: (**A**) stabilising membrane adaptation by *cis-trans*-isomerase (Cti); (**B**) release of outer membrane vesicles and concomitant cell surface alteration by removal of hydrophilic lipopolysaccharides (LPSs); (**C**) export of chemicals via efflux transporters (RND, resistance-nodulation-division transporter family; SMR, small multidrug resistance transporter family); (**D**) damage recovery, e.g. by chaperones (like ClpB and GroEL/ES) that support correct protein folding and assembly, or DNA repair via the SOS response; (**E**) aldehyde elimination via oxidation (oxidation states of carbonyl carbon indicated in red); (**F**) rerouting of energy and redox metabolism to provide reducing equivalents for response mechanisms inactivating reactive oxygen species (ROS) and energy-demanding tolerance mechanisms. While vesiculation and chaperone-aided protein folding are ubiquitous stress responses in Gram-negative bacteria, *cis-trans*-isomerisation is a rather specific feature of Pseudomonads. Here, redox and efflux transporter equipment differs in individual strains. CM, cytoplasmic membrane; OM, outer membrane.

A key feature of *Pseudomonas* strains making them so interesting for biotechnological applications is their inherent stress response and resistance to a variety of stress parameters, including antibiotics and solvents. In particular, solvent resistance has been extensively investigated. Solvents and other (hydrophobic) chemicals mainly affect the cell envelope by accumulation in the outer membrane or crossing it via porins, thus reaching the cytoplasmic membrane [23,24]. This can lead to the loss of essential membrane functions (cell integrity, enzymatic matrix, barrier for diffusion and electrochemical gradients) due to impaired membrane stability [25]. To maintain a specific degree of membrane fluidity, bacteria can adapt their lipid composition by biosynthesis of saturated lipids [26]. Besides, *Pseudomonas* species bear two powerful short-term adaption mechanisms [27] (Figure 1A,B).

A characteristic and almost unique adaptive response to environmental stress is the conversion of *cis* unsaturated membrane fatty acids into their corresponding *trans* configuration [28,29]. This very fast process neither consumes ATP nor involves cofactors [30] and is also independent of *de novo* protein and fatty acid biosynthesis. The periplasmic enzyme *cis-trans-*isomerase (Cti) converts the fatty acid residues palmitoleic acid (C16:1Δ9*cis*) and *cis*-vaccenic acid (C18:1Δ11*cis*) of phospholipids in the cytoplasmic membrane [28,29], leaving the position of the double bond unaltered. The shift from *cis-* to *trans-*fatty acids reduces membrane fluidity [23]. The gene for Cti (PP_2376 for *P. putida* KT2440) is present in all so far listed 34 *Pseudomonas* genomes in the Pfam database and could even be applied as a helpful molecular marker for the upcoming reorganisation of the genus (http://pfam.xfam.org/family/PF06934). This feature contributes to their inherent robustness [31]. Further engineering of this feature to improve bioproduction has not been described, although it might be thinkable: In *Escherichia coli*, expression of *cti* improved tolerance and carboxylic acid production [32], and *Pseudomonas* strains with additional copies of *cti* showed an increase in the level of *trans*-fatty acids [33].

In addition to rapid alteration in the phospholipids, Pseudomonads, like all Gram-negative bacteria, use vesiculation as a defence mechanism against various chemical (and non-chemical) stresses [34]. They release outer membrane vesicles (OMVs) with a diameter between 20 and 500 nm into the extracellular space, thereby altering the composition of the lipopolysaccharide (LPS) layer, the most distal part of their bacterial cell envelope. OMV formation and its numerous functions have been reviewed in detail [27,34–37]. The fast release of OMVs plays a major role in stress response, leading to a more hydrophobic bacterial surface and thus enhancing biofilm formation [38]. Bacteria living in biofilms or microcolonies are significantly more tolerant to antibiotics, solvents, and other forms of environmental stress [38,39], and this enhanced tolerance can be exploited in biofilm biocatalysis [40]. Furthermore, OMVs were shown to be crucial for unlocking otherwise unavailable polymeric substrates by exporting ligninolytic enzymes [41]. Vesiculation was also described to be naturally associated with the bacterial export of secondary metabolites like prodigiosin [42], the *Pseudomonas* quinolone signal (PQS) [43], and violacein [44], where OMVs may function as delivery vehicles. The mechanism could similarly support biotechnological production processes and even facilitate downstream processing. Vesiculation may even shield cells from a full toxicant dose, as OMVs were suggested to serve not only as an extracellular reservoir for a bacterial product but also as a non-cellular target [37,45]. The genetic basis of OMV formation is not yet entirely understood but could be connected to a range of genes [37]. Such knowledge has already been successfully applied for the engineering of hypervesiculation to support cell viability under stress conditions [46] or protein secretion in *E. coli* [47], but has not yet been utilised in the context of optimising *Pseudomonas* biotechnology and will certainly require fine-tuning to avoid extensive loss of lipids and membrane integrity [48,49]. The above-mentioned urgent response mechanisms enable *Pseudomonas* species to react quickly to emerging adverse conditions, making them robust candidates for whole-cell biotransformation processes and accessing new-to-nature chemistry.

### Chemical export machinery avoids intracellular accumulation of toxicants

The active extrusion of molecules plays a major role in the resistance of bacteria towards various toxic compounds because the invasion of toxic chemicals cannot be prevented completely by cell envelope stress response mechanisms (Figure 1C). Implicated efflux transporters are structurally and mechanistically diverse [50] and are categorised into different families [51] (Table 1).

**Table 1:** Resistance-mediating extrusion transporters in *Pseudomonas*

Name	Superfamily	Substrate(s)^*^	Representative host(s)	References
TtgABC, ArpABC, MexAB-OprM	RND	Antibiotics, heavy metals, mono- and polycyclic aromatics, short- and long-chain alcohols, polyphenols (e.g. naringenin, quercetin, phloretin), monoterpenoids, bipyridyls	*P. putida* KT2440, DOT-T1E, S12, GS1 *P. taiwanensis* VLB120 *P. aeruginosa* PAO1 *P. syringae* B728a	[52–58,60,61,70,71]
TtgDEF	RND	Aromatic solvents (i.e. toluene and styrene), monoterpenoids (i.e. geraniol), long-chain alcohols	*P. putida* DOT-T1E, GS1	[55,61,71]
TtgGHI, SrpABC	RND	Mono- and polycyclic aromatics (e.g. toluene and styrene, biphenyls), long-chain alcohols	*P. putida* DOT-T1E, S12 *P. taiwanensis* VLB120	[55,56,59,70,71]
MexCD-OprJ	RND	Antibiotics, polyphenols (i.e. phloretin), triclosan, acriflavine, alkaloids (i.e. berberine)	*P. aeruginosa* PAO1 *P. syringae* B728a	[54,72]
MexEF-OprN	RND	Antibiotics, polyphenols (i.e. phloretin), triclosan, alkaloids (i.e. berberine), formaldehyde^†^, glycolaldehyde^†^, vanillin^†^, 2,2-bipyridyl	*P. putida* KT2440 *P. aeruginosa* PAO1 *P. syringae* B728a	[54,62,72–75]
MexHI-OpmD	RND	Phenazines (i.e. 5-methylphenazine-1-carboxylate), antibiotics	*P. aeruginosa* PAO1, PA14	[65,67]
ParXY-TtgC MexXY-OprM	RND	Antibiotics	*P. putida* KT2440 *P. aeruginosa* PAO1	[53,76]
TtgK	MFS	Toluene	*P. putida* DOT-T1E	[77]
PP_1271-73^‡^	MFS	4-Hydroxybenzoate, vanillin^†^, 3-chlorobenzoate^†^, propionate, toluene^†^	*P. putida* KT2440, S12	[66,68,69,75,78]
PP_3349^‡^	MFS	Formaldehyde^†^	*P. putida* KT2440	[73]
PP_3658^‡^	MFS	Formaldehyde^†^	*P. putida* KT2440	[73]
Psyr_0228^§^	MFS	Antibiotics	*P. syringae* B728a	[72]
Ttg2ABC	ABC	Antibiotics, toluene, *p*-coumarate, heavy metals, *tert*-butyl hydroperoxide	*P. putida* KT2440, DOT-T1E	[15,77]
Psyr_0541^§^	SMR	Antibiotics, alkaloids (i.e. berberine)	*P. syringae* B728a	[72]
EmrE	SMR	Antibiotics	*P. aeruginosa* PAO1	[79]
NorM_PS	MATE	Antibiotics, 4′,6-diamidino-2-phenylindole	*P. stutzeri* ATCC 14405	[80]

This table provides an overview of the most important efflux transporters, their superfamily, substrates, and hosts. It does not provide a complete list of efflux transporters. Abbreviations: MFS, major facilitator superfamily; RND, resistance-nodulation-division transporter family; SMR, small multidrug resistance; ABC, ATP-binding cassette; MATE, multidrug and toxic compound extrusion.

* Substrates are representative as many transporters have a broad substrate range. Substrate spectrum can differ between representative hosts.

† Suggested substrate due to a responsive up-regulation of the transporter; an actual contribution to resistance was not investigated.

‡ Transporter name not available; locus tag of *P. putida* KT2440 used as reference.

§ Transporter name not available; locus tag of *P. syringae* B728a used as reference.

In Pseudomonads, efflux transporters of the resistance-nodulation-division (RND) family belong to the critical repertoire conveying resistance towards a broad spectrum of toxicants including antibiotics [52,53], biocides [54], heavy metals [51], mono- and polycyclic aromatics [55–59], short- and long-chain alcohols [59,60], (cyclo-)alkanes [59], monoterpenoids [61], and aldehydes [62]. Several different RND efflux pumps are present in *Pseudomonas* [63], however, strains commonly used in biotechnological applications differ in their equipment, which may have direct implications on their suitability as workhorses for specific product categories (Table 1).

The efflux pumps TtgABC, TtgDEF, and TtgGHI were thoroughly studied in the past and they all contribute to toluene resistance in *P. putida* DOT-T1E [55]. Unlike TtgDEF and TtgGHI, TtgABC belongs to the core genome of *P. putida* [64] and is especially relevant for the extrusion of antibiotics. TtgDEF is genetically co-localised with a toluene degradation cluster, extrudes toluene and styrene, and is present in *P. putida* DOT-T1E and *P. putida* GS1 [18,61]. TtgGHI is a key determinant regarding resistance towards aromatic solvents such as toluene and styrene, enabling growth in the presence of a second phase of these solvents [18,59]. Consequently, strains harbouring this efflux pump are considered to be solvent-tolerant, while the absence of TtgDEF and TtgGHI in *P. putida* KT2440 renders this strain sensitive to solvents.

In addition to RND efflux transporters, ATP-binding cassette (ABC), major facilitator superfamily (MFS), multidrug and toxic compound extrusion (MATE), and small multidrug resistance (SMR) transporters were also suggested or shown to be involved in resistance to a wide variety of biotechnologically interesting stressors in *Pseudomonas* (Table 1). However, only a few representatives have been identified and sufficiently characterised. Thus, for many, the substrate spectrum has not been (fully) revealed, yet.

The extrusion of toxicants is likely an ancient mechanism relevant for *Pseudomonas* (and other bacteria) in their ecologic niche, e.g. to resist plant antimicrobials and natural antibiotics [51] or to enable growth on natural petroleum seeps. The large diversity and broad substrate spectrum of efflux transporters enabled an adaptation to many different anthropogenic substances including synthetic antibiotics [65], solvent contaminants [55], and halogenated aromatics [54,66]. Furthermore, efflux transporters are not only important for the export of exogenous toxicants but can also extrude natively produced molecules such as phenazines [67] or heterologous products such as *p*-hydroxybenzoate [68] and propionate [69]. Besides, the extrusion of solvents also increases the degree of freedom regarding the extractant selection in biphasic fermentation for *in situ* removal of toxic substrates or products and can thus alleviate the toxic effect of compounds that are not subject to efficient extrusion themselves [19]. Therefore, the microbial production of bulk chemicals (e.g. butanol or phenol), high-value compounds such as flavonoids (e.g. naringenin) and alkaloids (e.g. berberine), or even new-to-nature compounds could benefit from the broad substrate spectrum of efflux transporters in the future.

### Dealing with the toxicant: detoxification and damage recovery

In addition to keeping or bringing toxicants out of the cells, bacteria also prevent or repair damage caused by a stressor (Figure 1D). For instance, DNA damage activates the bacterial SOS system, which maintains DNA integrity and replication via high- and low-fidelity repair mechanisms [81]. Mutants deficient in DNA repair genes, thus, show higher sensitivity to reactive compounds [82]. The up-regulation of chaperones from the heat shock protein families, like GroEL/ES or ClpB, is a ubiquitous response in bacteria to any kind of proteotoxic stress of physical or chemical nature [83,84], including the presence of solvents or aldehydes [62,85]. Accordingly, chaperone overexpression in *P. putida* improved tolerance to reactive wastewater components and enabled the valorisation of those compounds [62,84].

Furthermore, Pseudomonads are especially well-equipped to eliminate reactive and thus harmful compounds via conversion by a large set of redox enzymes (Figure 1E). Aldehydes, including the ‘sleeping giant of sustainable chemistry’ 5-hydroxymethylfurfural (HMF) [86] or the flavour compound vanillin, bear especially high potential to do harm as they are very reactive molecular species towards a plethora of nucleophiles, such as amino- or thiol- functionalities in, e.g., proteins or DNA [62]. Pseudomonads can rapidly convert toxic aldehydes into less noxious alcohol or acid derivatives. In contrast to *E. coli*, which primarily reduces aldehydes [87], Pseudomonads almost exclusively rely on oxidative deactivation [88]. This is of particular interest in the context of biotechnological processes where alcohols and aldehydes are to be oxidised into corresponding acids, such as the production of the plastic monomer 2,5-furandicarboxylic acid (FDCA) from HMF [89–91] or conversion of the monoterpenoid geraniol into geranic acid [61].

In *P. putida* KT2440, the periplasmic pyrroloquinoline quinone (PQQ)-dependent alcohol dehydrogenases PedE and PedH can oxidise a wide range of alcohols and aldehydes [92]. Both enzymes are highly expressed in *P. putida* KT2440 even in the absence of aldehydes or alcohols [93]. This high metabolic preparedness suggests that *P. putida* regularly encounters such toxicants in its natural habitat. The use of periplasmic enzymes seems logical from a tolerance perspective, as it may prevent high concentrations of aldehydes in the cytoplasm. It should be noted that the *ped* cluster encoding the above-mentioned dehydrogenases is not well conserved among Pseudomonads, and strains like *P. taiwanensis* VLB120 completely lack the cluster (pseudomonas.com [94]). However, Pseudomonads have a large repertoire of other dehydrogenases, and recent studies point to the involvement of, e.g., molybdenum-dependent enzymes [95–97].

The *Pseudomonas* redox equipment is also of particular interest for metabolisation of alternative substrates: Oxidases catalyse the first steps in the utilisation of alcohols as carbon source, including plastic monomers like ethylene glycol and 1,4-butanediol [93,98,99]. The targeted overexpression of glycolate oxidase was employed to avoid accumulation of the toxic intermediates glycolaldehyde and glyoxal during ethylene glycol metabolisation [99]. Similarly, *Pseudomonas* oxidation capacity was enhanced to increase FDCA production by co-expression of recombinant oxidases [91]. Such biotechnological oxidations require very high specific activities, with correspondingly high demand on the electron transport chain to regenerate redox cofactors. This, and also the use of true oxidases that produce H_2_O_2_, impose significant oxidative stress, which Pseudomonads are also well-equipped to handle [100] (Figure 1F). This feature is connected with a metabolic architecture geared towards formation of NADPH, the metabolic currency to counteract oxidative stress, upon exposure to oxidative agents [101]. Notably, oxidation of alcohols or aldehydes may also be detrimental when they are the desired end product of synthetic pathways or occur as biosynthetic intermediates, which may require gene inactivation [96,102]. Thus, complete understanding of *Pseudomonas*’ extensive oxidative enzymatic repertoire is indispensable to gain insights into ‘tolerance-by-conversion’ mechanisms and to enable microbial catalysis of new substrates and products.

## Perspectives in tolerance and cell factory engineering

In this chapter, we discuss the present toolbox of methods for the identification of tolerance-associated genes and their utilisation for engineering next-generation *chassis* with enhanced tolerance. Beyond that, we highlight key emergent strategies for pathway fine-tuning to establish stable cell factories for the effective production of natural compounds and novel biochemicals.

### Identification and implementation of tolerance-related genes

Identifying the genetic background of tolerant phenotypes is of major interest for developing robust production strains for industrial applications. So far, various methods have been explored (Table 2).

**Table 2:** Approaches to exploring tolerance features

Aim	Approach	Examples	Application note	References
Identification of natural genetic tolerance determinants	Identify single key factors via untargeted loss/gain-of-function studies	Tn*5* transposon mutagenesis; expression of (meta)genomic library	Potential false positives in loss-of-function approach with production strain (biosynthesis potentially compromised)	[106,107]
Identification of natural genetic tolerance determinants	Identify single key factors via targeted function validation	Gene expression after screening of geno- and phenotypes	Can ultimately lead to an understanding of networks	[70,143]
Identification of natural genetic tolerance determinants	Reveal multifactorial networks by addressing each gene	Transcript-/proteomics; sequence screening; enrichment within TRMR library	Applicable for studies with production strains, except TRMR (biosynthesis potentially compromised)	[62,68,103,116]
Creation of new features	Adapt strain via untargeted tolerance-induction with stress	Enrichment of mutants during TALE	Relevant for new-to-*Pseudomonas* and new-to-nature products; rather only for exposure (in production strain, biosynthesis potentially compromised)	[98,113,114]

The most conventional method for linking bacterial phenotypes to genes appears to be random gene inactivation via transposon mutagenesis. Screening for loss of tolerance and the identification of the transposon integration site in such clones was foundational in identifying, e.g. solvent pumps [59,103–105]. In contrast, gain of tolerance can be utilised to identify respective factors in (meta)genomic libraries of tolerant bacteria using susceptible strains for expression [106,107]. Both strategies are powerful to identify single key factors for tolerance but miss complex interacting networks.

With the emergence of respective technologies, it became possible to study whole transcriptomes (via microarrays or RNA-Seq) or proteomes to reveal multifactorial responses to exposure or biosynthesis of the stressor [62,68,103]. Moreover, advanced sequencing technologies facilitated the comparative analysis of complete genomes from related strains with different phenotypes to deduce tolerance-associated genes by sequence homology analysis or matching conspicuous phenotypes with outstanding genes [70,108–110]. Furthermore, the massive progress in sequencing technology evoked a reinvigoration of adaptive laboratory evolution (ALE) [111]. The strategy involves exposing a bacterial strain to a sublethal concentration of the stressor, which is usually increased over subsequential cultivations. This enables the development of robust strains and the elucidation of the genetic background conveying the tolerance [111,112]. This approach, designated as tolerance ALE (TALE) [113], is straightforward but rather time-consuming in dependence on the basic growth rate and the speed of adaption [93,113,114].

To achieve high throughput, recent studies combined the selection methodology of ALE with the ‘targeting each gene’ concept of transposon libraries and tracked the population dynamics in mixed cultures exposed to stress to quantify which mutant strains were enriched [15,61,115]. Conceptually similar, trackable multiplex recombineering (TRMR) of a barcoded cassette with a strong promoter and ribosome-binding site (RBS) upstream of virtually every gene allowed quantification of each mutant in mixed culture under stress conditions [116]. This strategy has not yet been applied in Pseudomonads but was suitable, e.g., to identify several genes contributing to furfural tolerance in *E. coli* [116].

**Figure 2:**
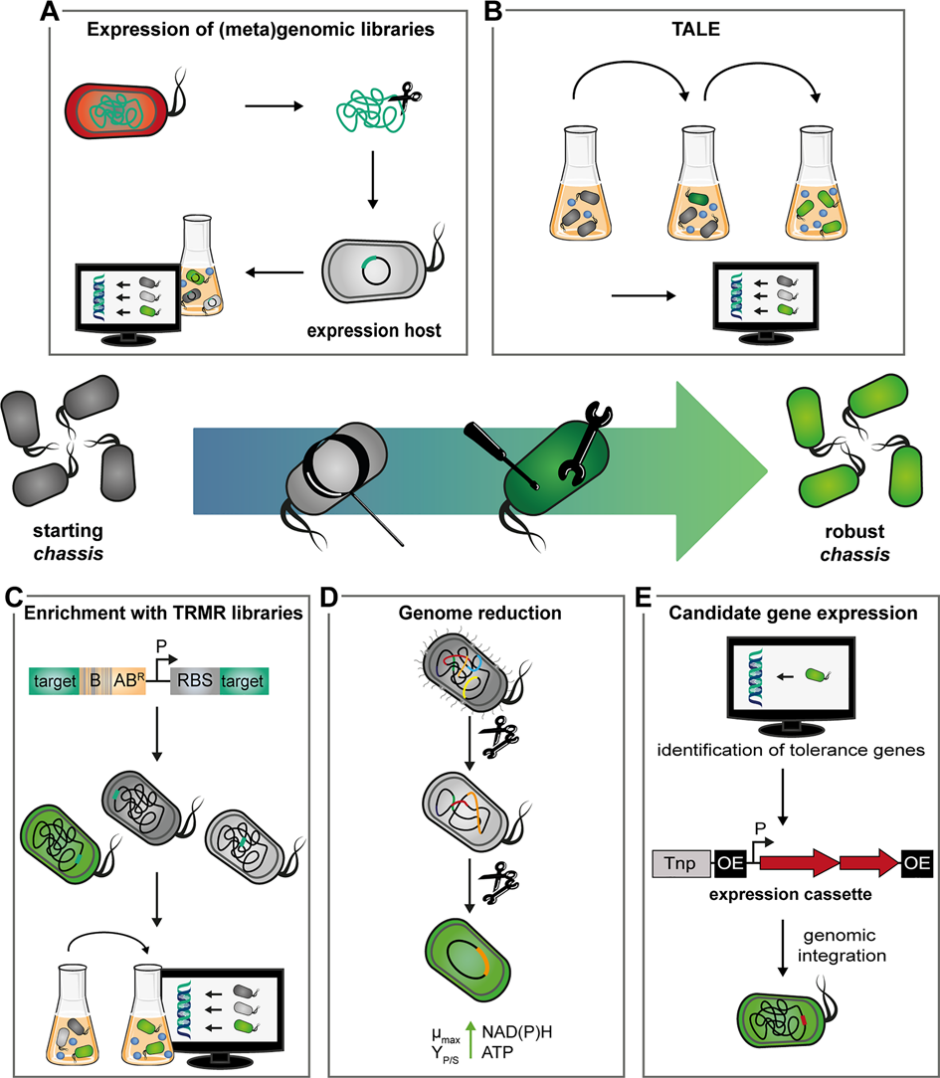
Engineering *Pseudomonas* strains with optimised tolerance Depicted are five approaches to building robust *chassis* (green cells) with tolerance to chemical stress (blue spheres). (**A**) (Meta)genomic libraries, e.g. of strains living in habitats with harsh environmental conditions, are promising to screen for tolerance-conveying genes by expression and exposure to a stressor. (**B**) In TALE, adaptive changes accumulate during long-term selection under stress-inducing conditions and can be identified by whole-genome sequencing. (**C**) TRMR facilitates the genomic integration of a DNA cassette with a sequence barcode for tracking (B), an antibiotic resistance gene (AB^R^), a strong promoter (P), and an RBS upstream of virtually every gene. Under stress conditions, the strains overexpressing genes that confer enhanced tolerance are specifically enriched within the barcoded library. (**D**) The removal of energy-intensive features during genome reduction can free capacities (in form of NAD(P)H and ATP) required to sustain tolerance, maximal growth rates and production yields. (**E**) Previously identified resistance-associated candidate genes are integrated into the genome of a ‘starting *chassis*’ by different genetic tools, e.g. a transposon (Tnp, transposase encoding region; OE, outside end of transposon).

Most of the described approaches are suitable for the elucidation of tolerance-associated genes and at the same time for the implementation of tolerance in a host (Figure 2). However, the typical methodology relies on exposure to externally applied stressors, although the actual motivation often was the efficient biotechnological production of the compound. Even though this might be sufficient for many membrane-permeable compounds [69], further studies directly focusing on production and the associated response [68] will be useful to identify tolerance-related genes with applicability in bioproduction. Notably, ALE-type strategies are of limited use for this approach, since stress-induced selection processes are likely to yield suppressor mutations that abolish biosynthesis of the xenobiotic stressor. Here, the use of ‘omics’ technologies appears favourable to uncover sensory and regulatory networks evoking multilevel responses to a production.

While major components of the *Pseudomonas* response to chemical stress could be identified (Figure 1), it is important to note that many of the mentioned studies suggest tolerance to rely on complex and highly diverse networks [81,117–119], meaning this feature is generally not trivial to transfer from one organism to another. Moreover, the formation of resistance determinants such as multicomponent efflux pumps, which have to be operated constantly to effectively lower the intracellular concentration of a toxicant, is associated with a high energy demand which can lead to reduced biomass yield, productivity, and even in some cases reduced tolerance [19,25,120–123]. It is therefore not practical to aim at creating one fully robust strain that can cope with any kind of stressor. For biotechnological use, it appears more attractive to isolate uniquely adapted [124–126] or to create new strains with distinct properties enabling the *à̀ la carte* selection of the most appropriate *chassis* for a specific product or process [120].

Since so far, descriptions of the targeted engineering of *Pseudomonas chassis* with optimised resistance for effective biotechnological applications are scarce [62,69,99,120,127], conclusions about generally successful strategies for strain development cannot yet be drawn. Based on studies with other chassis [85,128–130], we propose that due to the high metabolic costs, removal of dispensable energy-intensive cellular features [120], along with fine-tuning the expression of the tolerance-conveying gene(s), is essential for the construction of an optimal biotechnological cell factory. The latter is important for efflux pumps, where too strong expression can destabilise the membrane [60]. This fine-tuning can be achieved through controllable and stable gene expression. For *Pseudomonas*, a range of well-characterised, adjustable expression systems is available including inducible promoters with low leakiness (e.g. XylS/*Pm*, RhaRS/*P_rhaBAD_*, AntR/*P_antA_*, AraC/*P_araB_*) [131–133], or synthetic libraries of constitutive promoters with defined activities [132,134]. The use of plasmids for gene introduction enables rapid construction of different strains [135]. Nevertheless, genomic integration is usually preferable for avoiding problems such as plasmid instability or plasmid-related growth impairment [136–138]. For chromosomal integration, different genetic tools are available and already summarised in previous reviews [3]. A distinction can be made between randomised (e.g. via transposon Tn*5*) [139,140] and site-specific integration. Especially prominent here are transposon Tn*7* [132,134] and homologous recombination facilitated by recombinases (e.g. RecET) [141]. Counterselection strategies for genomic modifications including gene deletions have also been successfully adapted to *Pseudomonas* (e.g. by use of *sacB*, CRISPR-Cas9, or I-*Sce*I) [142]. This array of tools, together with an increasing understanding of tolerance traits, will facilitate the generation of *chassis* with specifically adapted tolerance features in the near future.

### Engineering of optimal flux in cell factories with novel pathways

**Figure 3:**
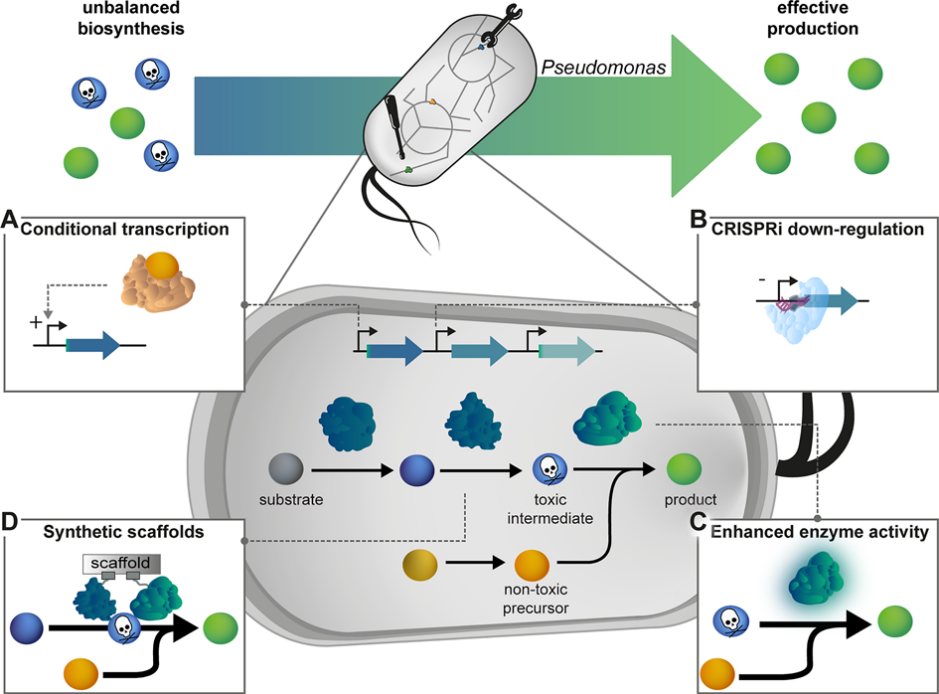
Key emergent strategies for pathway balancing At the metabolite level (spheres), an unbalanced biosynthesis can lead to accumulation of toxic intermediates in native or synthetic biochemical networks. Sophisticated regulation and balancing of metabolic fluxes towards and from the toxic intermediate can support effective production of targeted end-products. (**A**) Dynamic control via a transcription regulator activates the reactions leading to the toxic intermediate strictly under the condition that its reaction partner (orange sphere) is available at sufficient concentrations for effective conversion into the desired product. (**B**) Tight transcription control can be achieved by CRISPR-interference. (**C**) Enzymes with enhanced activity facilitate effective conversion and synthesis of the desired end-product. (**D**) Synthetic scaffolds increase local concentrations of pathway enzymes for effective substrate-channelling with decreased diffusion of intermediates.

The biosynthetic repertoire of *Pseudomonas* together with implementation of heterologous pathways allows the utilisation of various substrates and synthesis of highly diverse products such as aromatics, glycolipids, and terpenoids [2–4]. *De novo* biosynthesis of fluorometabolites from mineral fluoride has recently paved a new way to fluorinated building blocks [12]. An ideal cell factory for respective biotechnological applications is composed of a *chassis* that exhibits inherent or engineered tolerance to the involved substrate and product, and a metabolic pathway with optimal flux between the two. As discussed above, imbalances in a catabolic pathway can lead to accumulation of toxic compounds such as aldehydes that require elimination [99]. Also for anabolic recombinant pathways like multistep terpenoid biosynthesis, toxicity of biosynthetic intermediates is discussed as one major challenge for effective production [144]. Optimal balancing of intrinsic or recombinant pathways may thus become necessary, especially when the involved biocatalysts are confronted with non-natural substrates, affecting reaction rates and the overall catalytic efficiency (Figure 3).

Prominent catabolic routes in *Pseudomonas* exploited in both the context of bioremediation and the production of, e.g., muconic acid and derivatives thereof [145], are cleavage pathways involved in the degradation of various aromatic chemicals. Extensive studies on the *ortho*-cleavage pathway’s kinetics revealed that the overall flux is controlled by substrate uptake as well as by the enzymatic reactions forming and consuming the toxic intermediate catechol [146]. Bioproduction of novel building blocks derived from muconic acid requires an in-depth understanding of pathway regulation. For instance, when exposed to halogenated analogues of the pathway substrates, the cells accumulate halogenated catechols [147–149]. This phenomenon can be attributed to both a significantly reduced activity of the catechol-1,2-dioxygenases on substituted catechols [150] and a different (or lacking) response of the pathway regulators, preventing an efficient metabolic flux.

Protein engineering combined with a suitable screening assay can be a viable strategy to adapt a key enzyme towards accepting alternative substrates. Engineering of the prodigiosin ligase is a successful example of this [151,152]. As previously discussed, enhancing the expression level of a detoxifying oxidase was key for the utilisation of ethylene glycol [99]. In another case, accumulation of toxic glycolaldehyde as intermediate of installed xylose catabolism was avoided by deletion of a regulator leading to derepression of its conversion [127]. In both cases, adjusting the metabolic flow was thus key for the effective utilisation of both new-to-*Pseudomonas* substrates.

Balancing the expression of two biosynthetic genes was key to the production of bisdemethoxycurcumin in *P. putida* KT2440 to avoid the accumulation of undesirable intermediates and toxic effects [153]. Generally, the use of promoters with stringently adjustable expression strength and genome integration of expression cassettes appears favourable to achieve stable and controlled expression of biosynthetic genes, as discussed above in the context of tolerance-associated genes. Modular cloning methods allow efficient assembly and testing of various constructs [154–156]. However, adapting the expression of one gene to release enzymatic bottlenecks is not always a feasible option to avoid the effect of toxic intermediates. The kinetic parameters of certain enzyme–substrate combinations can be significantly inferior compared with other reactions within the same pathway, even at high concentrations of the catalyst. In these cases, the upstream enzyme levels need to be adjusted to avoid a metabolic imbalance at the bottleneck. Using multiple promoter systems, which can control expression of different genes independently, may be necessary [153,157]. Expression strengths can be further tuned through the use of translation initiation sequences of defined strengths, designed either with bioinformatic tools like the RBS Calculator for a given gene (https://salislab.net/software/predict_rbs_calculator) or standardised by balancing elements controlling transcription and translation initiation (bicistronic designs) [158].

Furthermore, a dynamic regulation approach can be employed to balance the biochemical activities based on metabolite concentrations: CRISPR-interference [159–161] as well as conditional proteolysis systems [162] have been implemented in *Pseudomonas* and can be used to down-regulate biochemical functions leading up to the toxic intermediate (e.g. by coupling the output to a suitable expression system, biosensor or riboswitch). For enhancing *E. coli*-based biodiesel production, where the desired product consists of precursors originating from two independent pathways that include a toxic intermediate, metabolite-inducible expression systems could be developed to allow a conditional activation of the toxicity-generating reactions only when the availability of the second precursor reached a threshold [163]. Aside from avoiding stress, such mechanisms are also ideal for avoiding unwanted intrinsic down-regulation of biosynthetic networks by key intermediates, which often occurs in biosynthetic pathways and impairs product yield. Another emerging strategy to achieve a higher and more balanced flux is to spatially organise enzymes within synthetic scaffolds [164,165] to create high local concentrations of metabolites and enzymes, with well-defined stoichiometry to support immediate conversion without diffusion of intermediates. All these strategies (Figure 3) together with an increasing understanding of tolerance traits will be instrumental for effective production applications and expanding the catalytic landscape of *Pseudomonas* towards novel chemistries [12,166].

## Conclusion

Proof-of-principle studies on the microbial production of many different compounds of interest are abundant in current literature. However, efficient high-level production of such compounds is relatively rare, because often the producer microbes typically applied to this end encounter challenging chemistries at multiple levels. Construction of robust cell factories will therefore be critical to take the next steps on the path towards sustainable bioproduction. Based on the current state of research, bacteria from the *Pseudomonas* clade represent especially promising *chassis* organisms in that respect. Here, unlocking new strains with unique tolerance features and knowledge-guided improvement of established *chassis* based on the biochemical characterisation of such traits will play an important role. This approach can enable the rapid construction of new synthetic hosts to finally establish a ‘*chassis à la carte*’ selection with specifically adapted tolerance features for any biotechnological application.

## Summary

- Efficient bioproduction of chemical compounds using whole cells as biocatalysts is often hampered by the limited ability of microbial hosts to cope with the stress linked to production.
- Pseudomonads are endowed with effective stress responses to many chemicals of biotechnological relevance.
- Understanding of the underlying tolerance mechanisms is increasing but their targeted exploitation and engineering is only just starting.
- A powerful molecular genetic toolbox will aid the construction of *chassis* with specific tolerance features to obtain optimised hosts for biotechnology.
- Pathway balancing strategies can further improve effective bioproduction of natural and new-to-nature compounds.

## Abbreviations

ABC: ATP-binding cassette (transporter family)
ALE: adaptive laboratory evolution
Cti: *cis-trans-*isomerase
FDCA: 2,5-furandicarboxylic acid
HMF: 5-hydroxymethylfurfural
HV1: host–vector (HV) system safety level 1
LPS: Lipopolysaccharide
MATE: multidrug and toxic compound extrusion (transporter family)
MFS: major facilitator superfamily (transporter family)
OMV: outer membrane vesicle
PQQ: pyrroloquinoline quinone
PQS: *Pseudomonas* quinolone signal
RBS: ribosome binding site
RND: resistance-nodulation-division (transporter family)
SMR: small multidrug resistance (transporter family)
TALE: tolerance ALE
TRMR: trackable multiplex recombineering
